# Honey bees and wild pollinators differ in their preference for and use of introduced floral resources

**DOI:** 10.1002/ece3.6417

**Published:** 2020-06-17

**Authors:** Christine Urbanowicz, Paige A. Muñiz, Scott H. McArt

**Affiliations:** ^1^ Department of Entomology Cornell University Ithaca NY USA

**Keywords:** alien plants, *Centaurea*, honey bees, introduced plants, invasive plants, knapweed, pollinators, preference

## Abstract

Introduced plants may be important foraging resources for honey bees and wild pollinators, but how often and why pollinators visit introduced plants across an entire plant community is not well understood. Understanding the importance of introduced plants for pollinators could help guide management of these plants and conservation of pollinator habitat. We assessed how floral abundance and pollinator preference influence pollinator visitation rate and diversity on 30 introduced versus 24 native plants in central New York. Honey bees visited introduced and native plants at similar rates regardless of floral abundance. In contrast, as floral abundance increased, wild pollinator visitation rate decreased more strongly for introduced plants than native plants. Introduced plants as a group and native plants as a group did not differ in bee diversity or preference, but honey bees and wild pollinators preferred different plant species. As a case study, we then focused on knapweed (*Centaurea* spp.), an introduced plant that was the most preferred plant by honey bees, and that beekeepers value as a late‐summer foraging resource. We compared the extent to which honey bees versus wild pollinators visited knapweed relative to coflowering plants, and we quantified knapweed pollen and nectar collection by honey bees across 22 New York apiaries. Honey bees visited knapweed more frequently than coflowering plants and at a similar rate as all wild pollinators combined. All apiaries contained knapweed pollen in nectar, 86% of apiaries contained knapweed pollen in bee bread, and knapweed was sometimes a main pollen or nectar source for honey bees in late summer. Our results suggest that because of diverging responses to floral abundance and preferences for different plants, honey bees and wild pollinators differ in their use of introduced plants. Depending on the plant and its abundance, removing an introduced plant may impact honey bees more than wild pollinators.

## Graphical abstract

We found that because of diverging responses to floral abundance and preferences for different plants, honey bees and wild pollinators varied in their use of introduced plants. In particular, knapweed (*Centaurea* spp.)—an introduced plant valued by beekeepers—was most often visited and preferred by honey bees but not wild pollinators. Our results suggest that when considering pollinator use of introduced plant species, it is critical to distinguish between honey bees—the dominant managed pollinator in the United States—and wild pollinators, as removing introduced plants may benefit one group of pollinators at a cost to the other.

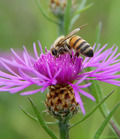

## 1 INTRODUCTION

There is a pressing need to ensure that declining pollinator populations have access to adequate foraging resources, which may include introduced plants (Bartomeus, Fründ, & Williams, 2016; Salisbury et al., 2015; Tepedino, Bradley, & Griswold, 2008). These plants represent a novel and sometimes abundant food source for pollinators (Bartomeus et al., 2016; Graves & Shapiro, 2003). However, in previous studies, some introduced plant species were avoided by pollinators (Chrobock, Winiger, Fischer, & van Kleunen, 2013), attracted fewer pollinator species than native plants (Moroń et al., 2009), or attracted honey bees more than wild pollinators (Morandin & Kremen, 2013). Few studies have looked across the entire plant community to identify whether and why introduced plant species as a group or individually are important in terms of pollinator visitation, resource acquisition, and pollinator diversity (but see Williams, Cariveau, Winfree, & Kremen, 2011). In this study, we first assessed community‐wide patterns of pollinator visitation rates and diversity on native and introduced plants. After finding that honey bees frequently visited and preferred knapweed (*Centaurea* spp.), an introduced plant in North America, we focused on this plant in the second part of our study. We quantified the degree to which honey bees collected knapweed pollen and nectar, and we identified wild pollinators visiting knapweed across New York.

Pollinator visitation and diversity on introduced plants are a function of field‐level floral abundance and trait‐based preferences (Williams et al., 2011; Wood, Kaplan, & Szendrei, 2018). Floral abundance may be positively correlated with pollinator visitation rate because pollinators are more likely to encounter and major on abundant species than rare species (Heinrich, 1979; Kunin, 1997). However, when the pool of available pollinators is saturated, highly abundant plants may have relatively low visitation rates (Essenberg, 2012; Totland & Matthews, 1998). Similarly, floral abundance may positively or negatively influence the diversity of pollinators visiting a plant, via more pollinator taxa encountering and majoring on abundant plants than rarer plants, or via competitive exclusion at highly visited plants reducing diversity (Hung, Kingston, Lee, Holway, & Kohn, 2019; Schaffer et al., 1983). In addition to floral abundance, pollinator decisions are influenced by preferences for certain plant traits. In a meta‐analysis, Morales and Traveset (2009) suggested introduced plants that outcompete native plants for pollinators are more attractive and may have larger flowers (Brown, Mitchell, & Graham, 2002; Moragues & Traveset, 2005) or more flowers per plant (Brown et al., 2002; Kandori, Hirao, Matsunaga, & Kurosaki, 2009). Furthermore, some introduced plants employ a super‐generalist pollination strategy, with pollen and nectar that are easily accessible to a large proportion of the pollinator community (Aizen, Morales, & Morales, 2008; Williams et al., 2011).

The western honey bee (*Apis mellifera*) may show fundamentally different visitation rates to introduced versus native plants compared with wild pollinators (defined here as non‐honey bee floral visitors). Our study takes place in North America, where honey bees are an introduced species. Here, honey bees may have trait‐based preferences for introduced plants due to the coevolution of species in their native range (Aizen et al., 2008; Stimec, Scott‐Dupree, & McAndrews, 1997). An association between honey bees and introduced plants may also be driven by floral abundance, with honey bees foraging on abundant introduced invasive plants to a greater extent than wild pollinators because of the honey bee's ability to recruit nestmates to abundant resources (Hung et al., 2019; Seeley, 1997). Most wild pollinators in North America are solitary bees and do not recruit nest mates (Hung et al., 2019). Additionally, honey bees more than other pollinators may seek out the highest per‐capita nectar and pollen rewards provided by some introduced plants (Aizen et al., 2008). These mechanisms may explain why honey bees were a dominant visitor to introduced plants in previous studies (Aizen et al., 2008; Wood et al., 2018) and have been linked to the spread of an invasive plant species (Barthell, Randall, Thorp, & Wenner, 2001).

Untangling how floral abundance and pollinator preference influence pollinator use of introduced plants could guide efforts to manage introduced plants while ensuring pollinators have access to adequate foraging resources. If pollinator visitation and diversity on introduced plants are primarily driven by abundance, then these plants could be replaced with native plants that provide similar quantities of pollen and nectar. In contrast, if pollinators prefer certain introduced plant species due to desirable traits, then land managers could consider maintaining these plants in the landscape if they are noninvasive (Salisbury et al., 2015). However, because honey bees and wild pollinators may differ in their use of introduced plant species, removing or maintaining introduced plants may benefit one group of pollinators more than the other. While beekeepers have opposed the removal of some introduced plants perceived as valuable to honey bees (Crane, 1981), the use of these plants by honey bees and wild pollinators is rarely quantified and needs to be considered. For example, beekeepers in New York are concerned that efforts to remove knapweed, which is prohibited and invasive in the state of New York (NYS DEC, 2014), would result in the loss of a critical late‐summer foraging resource (New York State Apiary Industry Advisory Committee, 2017). Knapweed has been found to be a major nectar and pollen source for wild bees and honey bees in Europe (Beil, Horn, & Schwabe, 2008; Steffan‐Dewenter & Tscharntke, 2000). However, it is unclear how often both honey bees and wild pollinators use knapweed resources relative to other late‐summer plants in North America.

In a two‐part study, we assessed the role of introduced plants as foraging resources for honey bees and wild pollinators. First, we used a pre‐existing, community‐wide dataset on pollinator visitation in three old fields in central New York. Across native and introduced plants, we determined how floral abundance and preference influenced (a) honey bee and wild pollinator visitation rates and (b) bee diversity. Because knapweed (*Centaurea s*pp.) was the introduced plant most often visited and preferred by honey bees, we also compared visitation rates of honey bees and wild pollinators to knapweed relative to all other coflowering plants. In the second part our study, we assessed knapweed pollen and nectar collection by honey bees and identified what insects visit knapweed across a broader survey of New York. Combined, our study provides a comprehensive analysis of the mechanisms and extent to which honey bees and wild pollinators use introduced versus native plants. This information can help managers understand how removing or maintaining introduced plants in a landscape may impact the pollinator community. Our focus on knapweed, which is currently being considered for expanded biocontrol in New York, provides an excellent real‐world case study.

## 2 METHODS

### 2.1 Part 1: Community‐wide analysis of pollinators visiting native and introduced plant species

We analyzed data on pollinator visitation rate and floral abundance of native and introduced plants in three open‐meadow old fields in central New York from May to September 2017. The three sites, named Lansing (Lat: 42°32′24.4932″N, Long: 76°29′47.9076″W), McDaniels (Lat: 42°32′11.5872″N, Long: 76°25′3.7668″W), and Whipple (Lat: 42°29′23.6328″N, Long: 76°25′49.818″W), were at least 5 km apart and partitioned into 9 to 11 ~ 100 × 100 m zones, depending on field size. Zones were used to randomly select areas of the field to survey plants and pollinators (below). The dataset used in Part I was originally collected as part of a large study characterizing plant–pollinator–pathogen networks (Graystock et al., In press); we used the data to test the relative importance of introduced plants for pollinators across an entire plant community.

#### 2.1.1 Floral visitation

Each site was surveyed once per week to record visitation rates, and sites were surveyed on separate days. At each site in each week, floral visitors were counted on 3–8 plant species, each species in a separate zone. On a typical observation day, three observers collected visitation data. Each observer randomly selected a 100 m × 100 m zone and haphazardly selected a plant species to observe among all flowering plants in the randomly selected zone. One goal of the larger study was to sample as many different plant species as possible, so individual observers avoided sampling the same plant twice on the same day when possible. The number of floral units being observed simultaneously in a small plot (approx. 1 m^2^) was recorded, with at least 10 floral units observed per observation period. A floral unit was defined as either an inflorescence or flower based on the scale that most pollinators foraged (Table S1). Ninety‐seven percent of observations were 15 min, and the remaining were 5 or 10 min due to logistical constraints. The number of visits by different insects was recorded, with the observer at least distinguishing between honey bees, other bees and wasps, flies, butterflies and moths, ants, and true bugs. We excluded ants and true bugs from our analysis and also excluded all visits occurring outside the flower, as it was unlikely these insects were foraging unless they were nectar robbing. We summed all remaining visits from all non‐honey bees, henceforth “wild pollinators.” Plants were identified to species except for *Centaurea* spp. (knapweed), which represented a mix of *Centaurea stoebe* and the *Centaurea jacea* s.l. complex (Gardou, 1972), both of which are introduced. We classified plant species as introduced or native according to the United States Department of Agriculture (USDA) native status (Table S1, https://plants.usda.gov/). We excluded one species with a native status listed as “both” on USDA (*Veronica serpyllifolia*). Of the remaining observations, each plant species was observed 1–16 times (median of 3) during the study. In total, there were 87 visitation observation periods across 24 native plant species and 122 visitation observation periods across 30 introduced plant species (see Table S1 for list of plant species).

#### 2.1.2 Bee diversity

During the same site survey used to record visitation rates, observers netted at 1–10 plant species, each species in a separate randomly selected zone. Because the larger study was primarily focused on bees, the main pollinator in our study system, observers only netted bees. For each netting period, all bees visiting flowers were collected on one plant species for 15 min , stopping the timer when placing bees in vials. Each plant species was netted 1–44 times (median of 4) during the study. In total, there were 137 netting periods across 27 native plant species and 194 netting periods across 26 introduced plant species (see Table S1 for list of plant species). Bees were identified to species except for 138 specimens (7% of the 1,924 total specimens; Table S2), which had missing or damaged identifying traits and were identified to genus or morphotype. Bees were identified using reference materials located in the Cornell University Insect Collection (CUIC: http://cuic.entomology.cornell.edu/). All identifications were conducted by P. Muñiz, taxa verifications were conducted by M. Arduser, and all voucher specimens are housed in the McArt lab or the CUIC. Bee diversity for each netting period was quantified using the Shannon diversity index and species richness (vegan package, Oksanen, 2019).

#### 2.1.3 Floral abundance

Each week, observers randomly selected three zones, randomly marked out a 10 m × 10 m area within each zone, and counted the total number of flowering stems of each species in this zone. With quadrats in three different zones, the study sought to capture plants that were rare or patchily distributed. For each species, we calculated average stem count per 100 m^2^ area per week at each site, averaging over the expanse of weeks that a flowering stem was observed. Therefore, each plant has one stem‐count estimate per site, which reflects how many flowering stems pollinators could encounter on average in a 100 m^2^ area. We did not calculate stem abundances on a weekly basis because a plant may have been absent in the randomly placed quadrats on any given week, which would be incorrectly noted as a complete absence from the site on that week. The number of floral units (inflorescences or flowers, as described in the floral visitation methods) per stem also was estimated during these site visits. Observers randomly selected one plant and counted the number of flowers on all stems of that plant. The exact number of flowers per stem was counted except for Asteraceae, for which observers counted 20–25 heads and used the area that those heads occupied to approximate the total number of flower heads on the plant. To capture more spatial and temporal variation in the number of floral units per stem, we supplemented these floral count estimates with the number of floral units counted on a total of 379 randomly selected stems across 56 plant species. These data were collected at each site approximately once every 2 weeks. During each site visit, 1–17 plant species were preselected based on what was flowering in the study area, and observers searched for these plant species in the site. Observers counted the number of floral units on 5–10 randomly selected stems. From these two sources of floral unit data, we calculated the average number of floral units per stem across all sites for each plant species. We had one estimate of floral units per stem per species across the entire study area rather than separate estimates for each site because of limited data per site. To arrive at site‐specific floral abundance estimates (floral abundance per 100 m^2^ per week), we multiplied the average weekly stem count at each site by the average number of floral units per stem for each species.

### 2.2 Analyses

#### 2.2.1 Influence of floral abundance on visitation rate

Across all flowering species, we tested how visitation rate varied with floral abundance, and whether the effect of abundance depended on native status (introduced or native) and pollinator type (honey bee or wild). To model visitation rate (visits per floral unit per minute), the number of visits during each observation period was used as the response, and log(floral units observed in observation period × minutes in observation period) was used as an offset in a generalized linear model with a negative binomial error distribution, which is an appropriate approach for modeling rates (O'Hara & Kotze, 2010; Reitan & Nielsen, 2016; glmmTMB function in glmmTMB package, Magnusson et al., 2019). Floral abundance (floral units per 100 m^2^ per week), native status, pollinator type, and their interactions were included as fixed effects. Observation period, site, week, and plant species were included as random effects. Observation period was included as a random effect because there were two visitation rates (one for honey bees and one for wild pollinators) for each observation period. Statistical significance here and below was assessed with likelihood ratio tests. In a post‐hoc analysis, we tested the significance of the four slopes, and we used pairwise comparisons with Tukey's correction to test for differences between slopes within a pollinator type (emtrends function in emmeans package, Lenth, Singmann, Love, Buerkner, & Herve, 2019). If the two slopes (visitation rate to native plants vs. floral abundance and visitation rate to introduced plants vs. abundance) were not significantly different within a pollinator type, we tested the main effect of native status within that pollinator type (emmean function).

#### 2.2.2 Preferences for introduced and native plants

We tested whether honey bees and wild pollinators showed preferences for native or introduced plants with a null‐model approach. We define preferred plants here as plants visited more often than would be expected by chance given their abundance relative to other plants observed in the same site in the same week. For each site visit, we scaled visitation rates by the proportional floral abundances of the plant species observed in that week and constructed 1,000 null matrices using the decimalr2dtable function in the bipartite package (Dormann, Fruend, & Gruber, 2019). This function randomly assigns visitation rates to plants but constrains marginal totals (i.e., maintains the sums of visitation rates for each pollinator type and plant species). Honey bees were not observed during 18 site visits, possibly because they recruited to floral hotspots elsewhere, such as nearby farms with mass‐flowering crops. Therefore, we did not calculate honey bee preference for plant species during these site visits. For these site visits, we used the same rules to generate 1,000 1‐dimensional null matrices for wild pollinators only. Preference was calculated as the observed visitation rate minus the mean expected visitation rate under the null model. Positive values indicated preference, and negative values indicated avoidance. We used the preference index in two ways. First, we calculated the average preference for each plant species and identified the species most preferred by honey bees or wild pollinators. Second, we modeled preference as a function of native status, pollinator type, and their interaction in a linear mixed‐effects model (lmer function, lme4 package, Bates et al., 2015). We included observation period, site, week, and plant species as random effects.

#### 2.2.3 Bee diversity

To test how 2017 bee diversity varied between native and introduced plants and with floral abundance, we separately modeled the Shannon diversity index and richness as a function of native status, floral abundance, and their interaction. The Shannon diversity index was modeled with a linear mixed‐effects model (lmer function, lme4 package), and richness was modeled with a generalized linear mixed‐effects model with a Poisson error distribution (glmer function, lme4 package). We included site, week, and plant species as random effects.

#### 2.2.4 Relative visitation to knapweed (*Centaurea* spp.)

We compared visitation rates by honey bees and wild pollinators to knapweed and coflowering plants. We analyzed 31 species across 98 observation periods during the knapweed flowering period (late July to mid‐August); knapweed was observed ten times. We modeled visitation rate with the same response variable, offset, random effects, and error distribution as the abundance analysis, above. Pollinator type (honey bee or wild) and plant type (knapweed or coflowering plant), and their interactions were included as fixed effects. We used pairwise comparisons with Tukey's correction to test for differences in visitation rates between groups.

### 2.3 Part 2: Pollinators using knapweed (*Centaurea* spp.) resources across New York

#### 2.3.1 Bee bread and nectar sampling

During the knapweed flowering period in 2018 (late July to mid‐August), we sampled hives from 22 apiaries across New York belonging to 14 beekeepers. Each apiary was sampled twice, approximately 2 weeks apart. Due to hives moving and colony loss within apiaries, we did not always sample the same hive twice. From one haphazardly selected hive in each yard, we collected fresh bee bread from 15 cells except in two cases, when we only found 12 cells of fresh bee bread. Fresh bee bread appeared chalky, light in color, and loosely compacted (Tsvetkov et al., 2017). Because ~80% of bee bread is consumed within 4 days of pollen collection (Anderson et al., 2014), our samples integrate several days of foraging. We also collected 5 ml of uncapped nectar from the same hive used to collect bee bread. Nectar and pollen samples were immediately placed on ice until they were transferred to a −20°C freezer.

#### 2.3.2 Knapweed pollen identification

We prepared bee bread slides using the palynological methods outlined in Urbanowicz et al. (2019). For the uncapped nectar samples, we subsampled 0.5 ml of nectar, added 1 ml of 40°C DI water, vortexed for 30 s, centrifuged for 10 min at 6K rpm, and decanted the supernatant. We repeated the process of adding water, vortexing, centrifuging, and decanting, and then added 1.5 ml 95% ethanol and vortexed for 1 min. We added 10 μl of the resulting suspension and 40 μl Calberla's solution to a glass slide. In approximately half of the nectar samples, we had to repeat the process of subsampling nectar and preparing slides until we counted 300 total pollen grains. At 400× magnification, a transect was initiated at a random location on each slide, and all pollen grains that were entirely in the field of view were classified as knapweed or other until a total of 300 pollen grains was reached. To identify knapweed pollen, we referred to knapweed pollen slides made from knapweed in central New York. We also referred to a pollen library collected in central New York (McArt, Fersch, Milano, Truitt, & Böröczky, 2017) to ensure knapweed was distinguished from other species. We calculated the average percentage of knapweed pollen in bee bread and nectar from each apiary and compared these percentages across apiaries.

#### 2.3.3 Pollinator survey

We sampled pollinators visiting knapweed (a mix of *C. stoebe* and *Centaurea jacea* s.l. species complex) in seven sites across New York during the knapweed flowering period in 2018. All 2018 sites were at least 24 km apart, and one 2018 site was located 500 m from a 2017 sampling site described in Part 1 (above). Sites were located along the roadside and opportunistically surveyed once in the afternoon when cloud cover was less than 50%. Insects foraging on knapweed were netted for 30 min, stopping the timer when placing insects in vials. Unlike 2017, when observers limited their collection to bees, we netted all foraging insects in 2018. Insects were identified to species except for *Lasioglossum* spp. and a female *Sphaerophoria* fly.

## 3 RESULTS

### 3.1 Part 1: Community‐wide analysis of pollinators visiting native and introduced plant species

We analyzed a total of 4,265 pollinator visits across 24 native plant species and 1,642 visits across 30 introduced plant species. Honey bees most often visited *Centaurea* spp. (introduced, mean visitation rate = 0.11 visits/floral unit/minute, mean floral abundance = 64 floral units/100 m^2^/week), *Lychnis flos‐cuculi* (introduced, 0.057, 19), and *Melilotus albus* (introduced, 0.037, 1,026). Wild pollinators most often visited *Rubus allegheniensis* (native, 0.19 visits/floral unit/minute, 366 floral units/100 m^2^/week), *Hypericum perforatum* (introduced, 0.19, 54), and *Rubus hispidus* (native, 0.15, 98). Table S1 provides the visitation rates and floral abundances of all plant species.

#### 3.1.1 Influence of floral abundance on visitation

**FIGURE 1:**
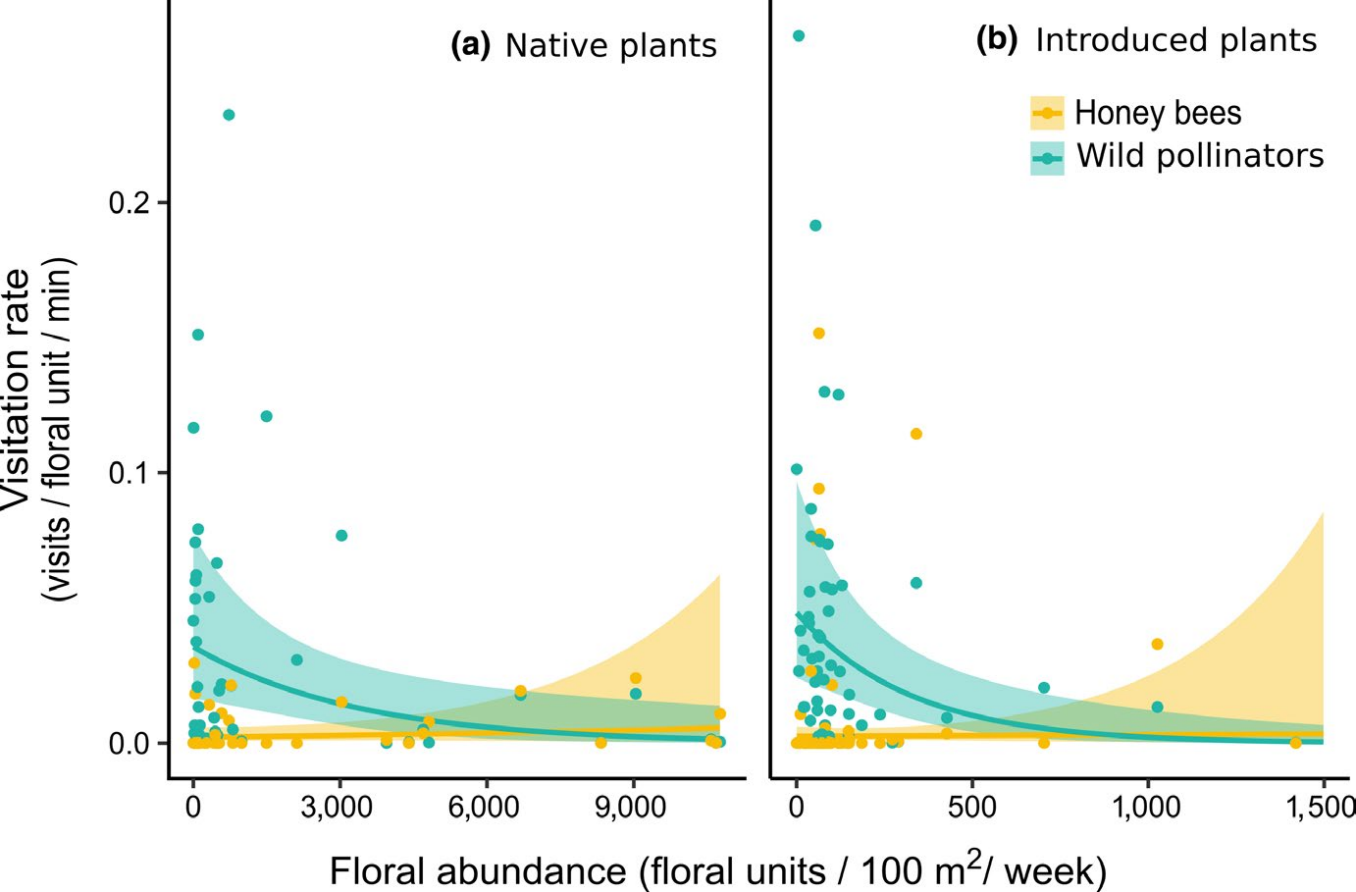
Association between floral abundance and visitation rate of honey bees and wild pollinators visiting native plants (a) and introduced plants (b). Points represent average visitation rates per plant species per site. Note that native plants had a higher maximum floral abundance than introduced plants

Visitation rate depended on the interaction between floral abundance, the native status of the plant species (native or introduced), and pollinator type (honey bee or wild; $\chi_{1}^{2}$ = 5.08, *p* = .024, Figure 1). Honey bees visited native and introduced plants at similar rates (post hoc Tukey's test: *p* = .83). The visitation rates of honey bees visiting both native (Figure 1a) and introduced plants (Figure 1b) were not significantly related to floral abundance (Tukey's test: *p* > .50 in both cases). In contrast, the visitation rate of wild pollinators visiting both native (Figure 1a) and introduced plants (Figure 1b) decreased with floral abundance (Tukey's test: *p* = .013 and *p* = .002, respectively). This decrease was steeper for introduced plants than native plants (Tukey's test: *p* = .027), meaning that wild pollinators visited native plants more often than introduced plants as floral abundance increased.

**FIGURE 2:**
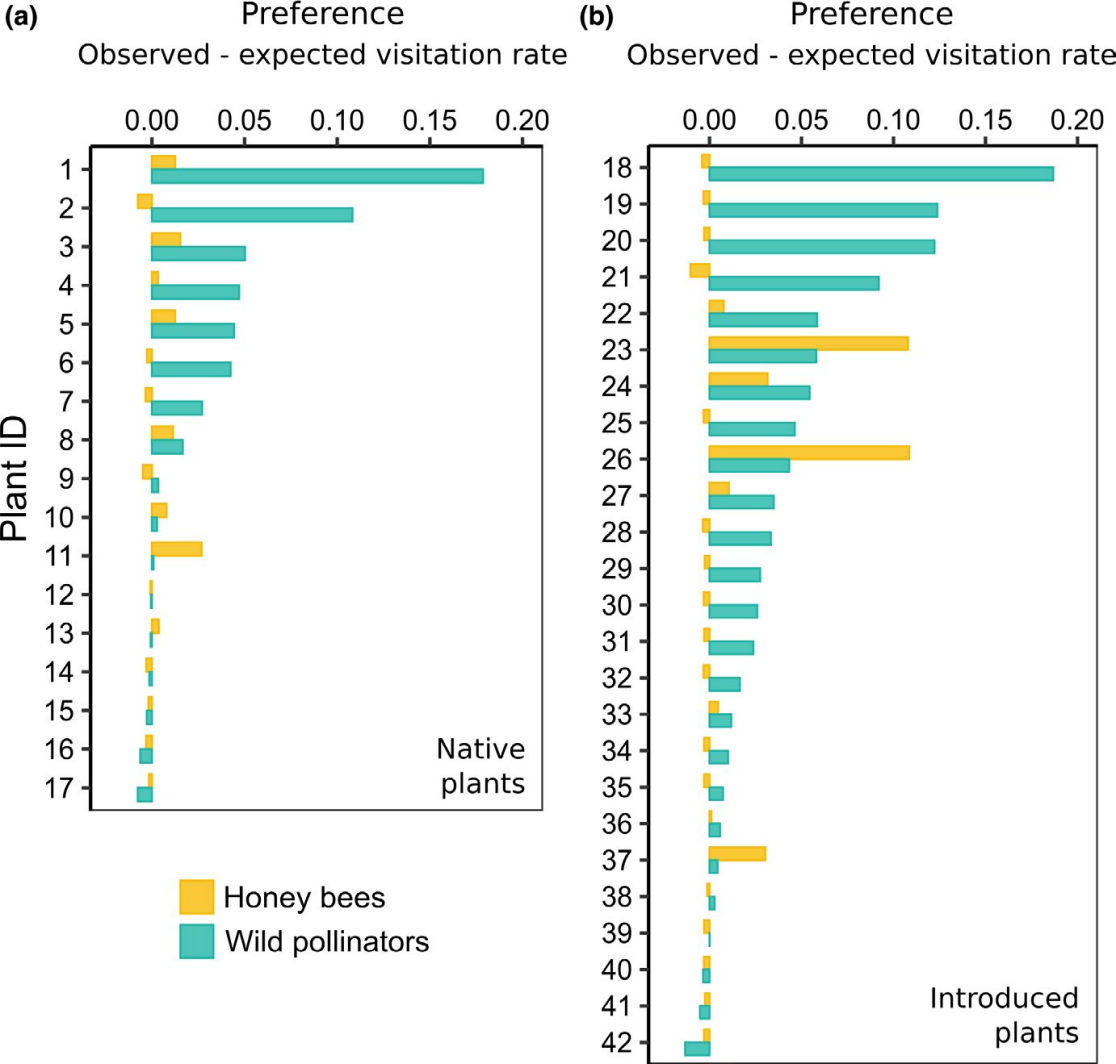
Average honey bee and wild pollinator preference indices for native plant species (a) and introduced plant species (b). Preference was calculated as the observed visitation rate minus the mean expected visitation rate under the null model. Positive values indicate preference, and negative values indicate avoidance. Each plant ID represents a separate species (listed in Table S1). Plants are ordered most to least preferred by wild pollinators. Only plants with preference information for both honey bees and wild pollinators are shown

Preferences for introduced and native plants*:* The majority of plants preferred by honey bees were also preferred by wild pollinators, but the order of preference varied between pollinator types (Figure 2, Table S1). Honey bees most preferred *Centaurea* spp. (introduced, mean preference index = 0.11), *L. flos‐cuculi* (introduced, 0.11), and *Cirsium arvense* (introduced, 0.032). Wild pollinators most preferred *H. perforatum* (introduced, mean preference index = 0.19), *R. allegheniensis* (native, 0.18), and *R. hispidus* (native, 0.15). Eighteen plants that were preferred by wild pollinators were avoided by honey bees (Figure 2). Table S1 provides preference indices of all plant species.

Neither honey bees nor wild pollinators showed a significant preference for native or introduced plants as a group (native status main effect: $\chi_{1}^{2}$ = 0.28, *p* = .59; native status × pollinator type interaction: $\chi_{1}^{2}$ = 0.39, *p* = .74). Honey bees avoided more plant species (fewer visits than expected by chance) than wild pollinators as a group, and so the average preference index of honey bees (0.019 ± 0.0061 *SE*) was lower than that the average preference index of wild pollinators (0.30 ± 0.0051) (pollinator type main effect: $\chi_{1}^{2}$ = 6.46, *p* = .011). During our observations, one plant species was never visited by wild bees, and 32 plant species were never visited by honey bees.

#### 3.1.2 Bee diversity

We analyzed 804 bees collected on 27 native plant species across 137 netting periods, and 1,120 bees collected on 26 introduced plant species across 194 netting periods. A total of 94 bee species were collected, and the most common species were honey bees (*A. mellifera*, 26% of bees), *Bombus impatiens* (16%), and *Ceratina mikmaqi* (6%). Bee diversity was highest on *R. allegheniensis* (native, mean Shannon–Wiener diversity per visitation period = 1.70, mean richness = 8.00 species), *Centaurea* spp. (introduced, 1.31, 6.11), and *Rosa multiflora* (introduced, 1.23, 4.00). Native plants as a group and introduced plants as a group did not differ in their diversity or richness of bees, and floral abundance did not explain diversity or richness (Table 1).

**TABLE 1:** Likelihood ratio test results for models relating bee diversity (Shannon diversity index) and richness to plant native status, floral abundance, and their interaction

Response	Source	χ^2^ (*df* = 1)	*p*
Diversity	Plant native status	0.38	.54
	Floral abundance	0.12	.73
	Plant native status × floral abundance	0.55	.47
Richness	Plant native status	0.16	.69
	Floral abundance	0.074	.79
	Plant native status × floral abundance	0.94	.33

#### 3.1.3 Relative visitation to knapweed (*Centaurea* spp.)

**FIGURE 3:**
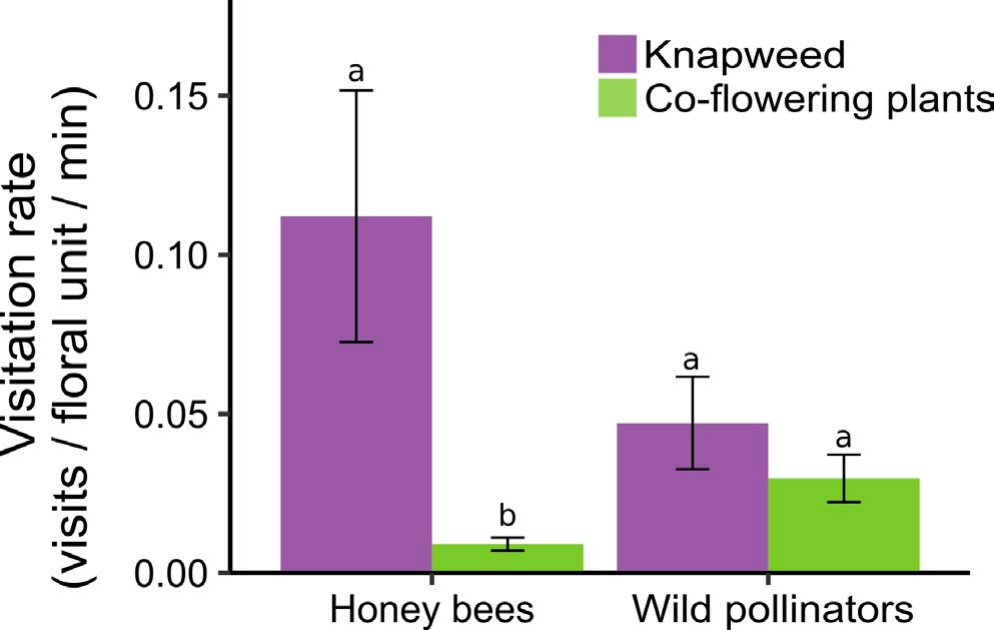
Mean (± standard error) visitation rates of honey bees and wild pollinators visiting knapweed (*Centaurea* spp.) and coflowering plants

Because of the high overall visitation rate and diversity of pollinators on introduced knapweed (*Centaurea* spp.), we assessed relative visitation rates of honey bees and wild pollinators. Honey bees but not wild pollinators visited knapweed at a significantly higher rate than coflowering plants (Figure 3, interaction: $\chi_{1}^{2}$ = 6.0, *p* = .014; Tukey's test for honey bees: *p* < .001; Tukey's test for wild pollinators: *p* = .89). The visitation rates of honey bees and wild pollinators on knapweed were not significantly different (Tukey's test: *p* = .62), meaning that, on average, honey bees visited knapweed at a similar rate as all other pollinators combined.

### 3.2 Part 2: Pollinators using knapweed (*Centaurea* spp.) resources across New York

#### 3.2.1 Knapweed pollen in bee bread and nectar

**FIGURE 4:**
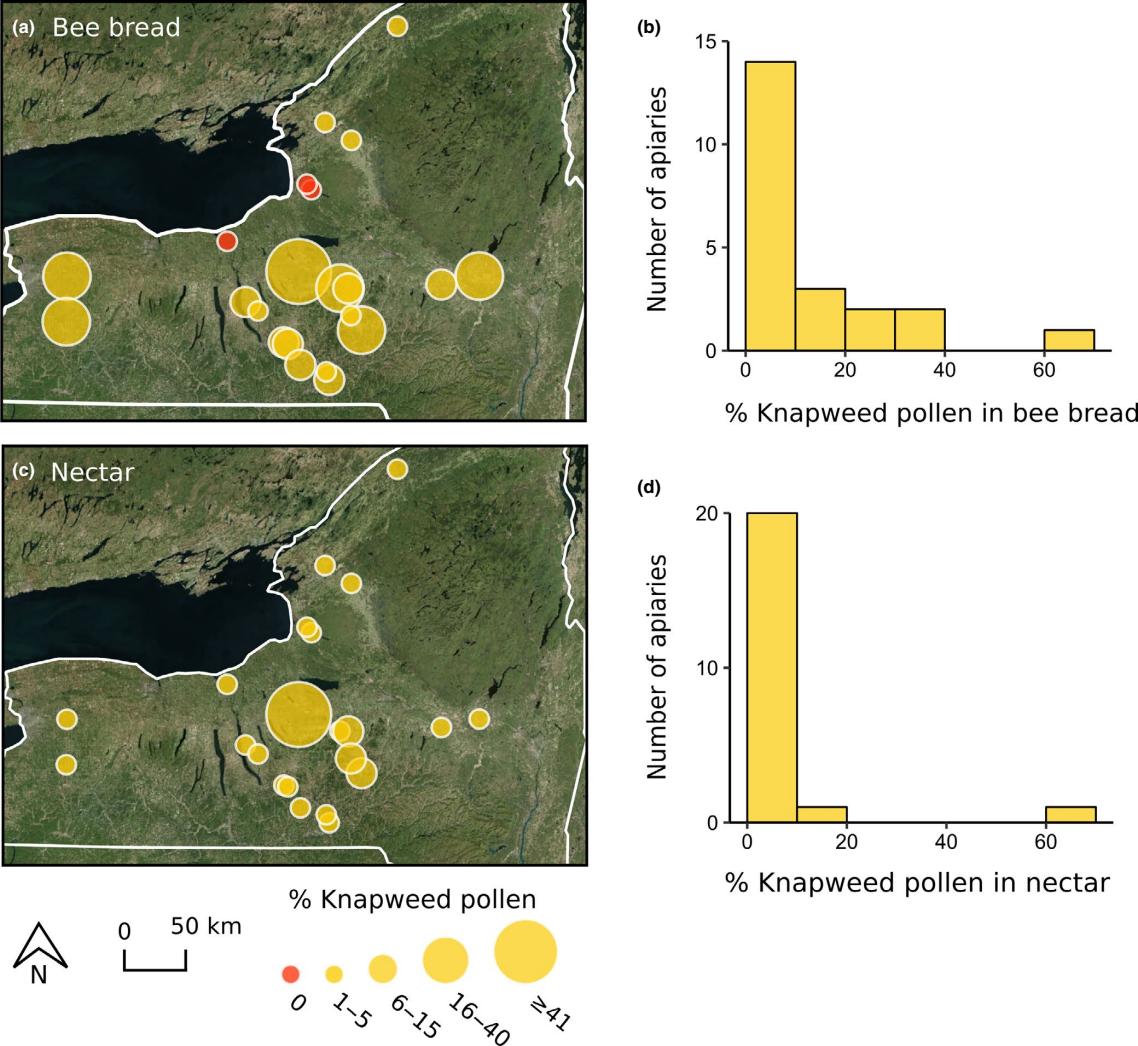
Percent knapweed (*Centaurea* spp.) pollen in fresh bee bread (a,b) and uncapped nectar (c,d) collected from 22 apiaries in New York

Hives in 86% of apiaries (19 of 22 apiaries) contained knapweed pollen in bee bread. Knapweed pollen comprised between 0% and 68% of bee bread pollen (median = 5.1%), with knapweed pollen absent in bee bread samples in three apiaries in northern New York (Figure 4a,b). Hives in all apiaries contained knapweed pollen in nectar. Knapweed pollen comprised 0.2%–63% of pollen in nectar (median = 1.3%; Figure 4c,d).

#### 3.2.2 Pollinator survey

Across seven knapweed sites, we collected a total of 446 pollinators representing 35 species. Honey bees were the dominant pollinator, composing 46% of all pollinators collected, followed by *B. impatiens* (8%) and *Ceratina calcarata* (7%; Table S3). Thirteen of the 21 bee species collected on knapweed in 2018 (3.5 total hours of netting on knapweed) were also collected on knapweed in 2017 (3.75 total hours of netting on knapweed, Part 1, above).

## 4 DISCUSSION

To understand the potential value of introduced plants to pollinators, we assessed the extent to which pollinators used introduced plants versus native plants across an entire plant community. Honey bees visited native and introduced plants at similar average rates regardless of floral abundance, suggesting honey bees can take advantage of highly abundant introduced plant species that they prefer. In contrast, wild pollinator visitation rate declined with floral abundance and did so more strongly for introduced plants than native plants, suggesting that wild pollinators prefer abundant native plant species over abundant introduced plant species. Furthermore, honey bees and wild pollinators preferred different introduced species. Across introduced plants, honey bees but not wild pollinators most preferred knapweed (*Centaurea* spp.), visiting it more frequently than coflowering plants and at a similar rate as all wild pollinators combined. Our survey of New York apiaries also showed that knapweed is a prevalent and, in some places, main source of pollen and nectar for honey bees in the late summer. Together, these results suggest that honey bees and wild pollinators differ in their use of introduced plants because of diverging responses to floral abundance and trait‐based preferences for different plants.

Honey bees and wild pollinators responded differently to floral abundance. The visitation rate of honey bees did not significantly change with floral abundance. Honey bees maintained visitation rates to highly abundant plants—both native and introduced —likely by recruiting to those plants in our field sites (Hung et al., 2019). Honey bees live in large colonies and are able to exploit abundant resources through communicating the location of these resources to nestmates. This recruitment of nestmates has been suggested as one reason that honey bees visit abundant invasive plants more frequently than wild pollinators (Morales & Aizen, 2006). Honey bees also visited rarer plants, perhaps because these plants were highly rewarding (Goulson, 1994), or honey bees may have been avoiding exploitative competition on abundant plants (Fontaine, Collin, & Dajoz, 2008).

Unlike honey bees, most wild pollinators in our system are solitary, do not recruit nestmates to abundant floral resources, and therefore did not maintain a constant visitation rate through recruitment of nestmates. Instead, wild pollinator visitation rate declined with floral abundance, likely because the pool of pollinators foraging on abundant flowers was saturated, with pollinators spread out over more flowers (Essenberg, 2012; Holzschuh et al., 2016). Interestingly, wild pollinator visitation rate declined more rapidly for introduced plants than native plants, which may indicate wild pollinator preference for abundant native plant species over abundant introduced plant species (Morandin & Kremen, 2013). This pattern was largely driven by the three most abundant introduced species (*Lythrum salicaria*, *M. albus*, and *Vicia cracca*), which also had low visitation rates. Consistent with these findings, our preference analysis also indicated that these three plant species were some of the most avoided plants by wild pollinators. It is possible that two of these plants in the Fabaceae family (*M. albus* and *V. cracca*) were avoided because pollinators were unable to open the corollas. One caveat when interpreting these results is that they represent the wild pollinator community as a whole. Visitation rates and preferences of wild pollinators likely vary between species, and visitation rates calculated for wild pollinators as a group mask this variation. The wild pollinator community in our study is representative of a typical temperate meadow system, dominated by small solitary bees, and our results are therefore biased toward the preferences and visitation rates of these bees. Overall, our results suggest that because honey bees can take advantage of abundant plants, maintaining abundant, preferred introduced plant species in the landscape may benefit honey bees. However, doing so may not benefit wild non‐*Apis*pollinators, which do not recruit to highly abundant plant species and which visited abundant native plants more than abundant introduced plants.

We found broad similarities and important differences between honey bee and wild pollinator preferences for specific plant species. Neither honey bees nor wild pollinators showed an overall preference for native plants as a group or introduced plants as a group, likely because traits influencing preference did not sort out between the two plant groups. This result agrees with the findings of Williams et al. (2011), who also found that pollinators did not generally prefer introduced plants over native plants. Fifteen of the 16 plants preferred by honey bees were also preferred by wild pollinators, suggesting that preferred plants are broadly attractive with accessible pollen or nectar. These plants included *Cichorium intybus*, *Cirsium arvense*, and *Centaurea* spp. (knapweed), which are considered pollination generalists and have been found to attract a large number of pollinators in other systems (Carson, Bahlai, Gibbs, & Landis, 2016; Orford, Murray, Vaughan, & Memmott, 2016). Among the preferred plants, however, honey bees and wild pollinators most preferred different plant species. For example, honey bees most preferred knapweed and visited knapweed ~13 times more frequently than coflowering plants. In contrast, knapweed was the eighteenth most preferred plant by wild pollinators, and wild pollinators visited it at a similar rate as coflowering plants. Competition between honey bees and wild pollinators may have also contributed to differences in relative visitation rates and apparent preferences, as high densities of honey bees have been shown to cause wild pollinators to switch to less rewarding plants (Magrach, González‐Varo, Boiffier, Vilà, & Bartomeus, 2017).

Across New York, we found knapweed pollen in the bee bread of 86% of apiaries and in the nectar of all 22 apiaries that we sampled; however, knapweed's contribution to bee bread and nectar varied considerably between apiaries. This variability is likely a reflection of the availability of knapweed within the foraging range of honey bees (Wood et al., 2018). Anecdotally, knapweed was an abundant plant in the apiary with the highest percent knapweed pollen in bee bread (67%) and nectar (63%). In contrast, we saw no knapweed plants in the three northern apiaries that had no knapweed pollen in their bee bread samples and low proportions (<2%) of knapweed pollen in their nectar. On average, we found less knapweed pollen in nectar than in bee bread, which was surprising because knapweed is valued as a nectar source by beekeepers (Maddox, 1979; Watson & Renney, 1974) and has relatively high volumes of nectar per flower (Hicks et al., 2016). Differences in the quantity of knapweed pollen in bee bread and nectar may be due to differences in foraging behavior of individual honey bees. For example, some forager bees specialize on nectar while others specialize on pollen (Winston, 1987). Furthermore, pollen can be over‐ or under‐represented in nectar depending on the plant species, and the proportion of knapweed pollen in nectar may not represent the volume of nectar that knapweed is contributing (Bryant & Jones, 2001).

Knapweed supported a relatively high diversity of bees in the community‐wide survey, and we found 42 pollinator taxa foraging on knapweed across New York. Bee diversity was not driven by abundance, suggesting that certain attractive traits, such as nectar volume and flower shape, are driving high pollinator diversity on knapweed. Carson et al. (2016) also found relatively high pollinator diversity on knapweed, with more pollinator species visiting knapweed than 12 native plants. However, in the same study, fields that were invaded and dominated by knapweed had lower overall pollinator diversitycompared to fields with a diversity of plant species, which supported pollinators before and after knapweed bloom. Similarly, fields invaded by goldenrod were found to have a lower diversity of plants and pollinators than noninvaded fields (Moroń et al., 2009). We therefore urge caution when interpreting how pollinator diversity may benefit from knapweed or other introduced plants in areas where introduced plants are invasive and replacing native plants.

The management of introduced plants requires addressing stakeholders with different priorities and assessing economic and ecological costs and benefits (Crane, 1981). For example, while natural area managers and ranchers seek to remove knapweed through biological control (Müller‐Schärer & Schroeder, 1993), beekeepers have opposed the removal of knapweed because it may be an important late‐summer foraging resource (Runk, 2010). Our results suggest that knapweed is a prevalent and sometimes locally important resource for honey bees in terms of being preferred and frequently visited for pollen and nectar. However, because knapweed can effectively invade plant communities and suppress native plant biomass and diversity (Herron‐Sweet, Lehnhoff, Burkle, Littlefield, & Mangold, 2016; Maron & Marler, 2008), maintaining knapweed in the landscape may come at an opportunity cost to wild pollinators that visit and prefer other plants more than knapweed, such as *Rudbeckia hirta* and *Monarda fistulosa*. Further research should focus on determining what traits are driving preferences for certain introduced plant species, including knapweed, and identifying similar native or noninvasive introduced species that could be used in restoration efforts.

## CONFLICT OF INTEREST

None declared.

## AUTHOR CONTRIBUTIONS

**Christine Urbanowicz:** Conceptualization (equal); Data curation (lead); Formal analysis (lead); Methodology (equal); Supervision (equal); Visualization (lead); Writing‐original draft (lead); Writing‐review & editing (lead). **Paige A. Muñiz:** Data curation (equal); Methodology (equal); Validation (equal); Writing‐review & editing (supporting). **Scott H. McArt:** Conceptualization (equal); Data curation (supporting); Formal analysis (supporting); Funding acquisition (lead); Methodology (equal); Supervision (equal); Writing‐original draft (supporting); Writing‐review & editing (equal).

## Supporting information

Table S1

Table S2

Table S3

## Data Availability

The data are archived in the Dryad Digital Repository. https://doi.org/10.5061/dryad.wh70rxwjz.
